# Space use and navigation ability of hens at housing in the aviary for the laying phase: effect of enrichment with additional perches and genotype

**DOI:** 10.1016/j.psj.2023.102962

**Published:** 2023-07-25

**Authors:** C. Ciarelli, G. Pillan, F. Bordignon, G. Xiccato, M. Birolo, A. Trocino

**Affiliations:** ⁎ Department of Agronomy, Food, Natural Resources, Animals and Environment (DAFNAE), University of Padova, 35020 Legnaro, Padova, Italy; † Department of Comparative Biomedicine and Food Science (BCA), University of Padova, 35020 Legnaro, Padova, Italy

**Keywords:** laying hen, enrichment, space use, animal distribution, collision

## Abstract

The present study tested the hypothesis that increased availability of perches could favor the adaptation and navigation ability of pullets of different genotypes at housing in a new aviary system for the laying phase. To this purpose, 900 Lohmann White-LSL and 900 Hy-line Brown were randomly allocated at 17 wk of age in 8 pens of an experimental aviary, according to a bifactorial arrangement with 2 genotypes (Brown vs. White) × 2 types of pens (enriched or not enriched with additional perches besides those of the aviary). Data collected between 17 and 20 wk of age showed that the enrichment with additional perches decreased the use of the aviary perches while the rate of successful landings/take-offs was unaffected. As for the effect of genotype, during the night a lower rate of hens on the floor (0.15 vs. 6.63%) and a higher rate of hens on the additional perches (2.47 vs. 0.98%) was found in White compared to Brown hens (*P* < 0.001); the former hens also used the third tiers for sleeping on the aviary uppermost perches (*P* < 0.001). During the day, White hens used more the third tier (32.8 vs. 15.6%; *P* < 0.001) and the additional perches (3.88 vs. 0.91%; *P* < 0.01) compared to Brown hens, while they stood less on the floor (18.3 vs. 22.6%; *P* < 0.05). White hens performed a significantly higher number of landings (80.7 vs. 21.9; *P* < 0.001) and of take-offs (74.3 vs. 10.0; *P* < 0.001) per pen compared to Brown hens. The risk of unsuccessful landings was higher in Brown compared to White hens (odd ratio: 6.65; 95% confidence interval: 4.36–10.1; *P* < 0.001). In conclusion, the enrichment with additional perches played a major role in hen distribution and space use than in their navigation ability. At the same time, the significant differences between the 2 genotypes call for a careful evaluation of the aviary design and animal management to optimize welfare at housing and possibly productive results of laying hens.

## INTRODUCTION

In Europe, cage-free systems for laying hens are going to fully replace all kinds of caged housing system since they can increase animal welfare by providing them with space for free navigation and areas, besides materials for specie-specific behaviors, such as nests for laying eggs, litter for dust bathing, and perches for vigilance and roosting (Hemsworth and Edwards, 2020; EFSA AHAW Panel (EFSA Panel on Animal Health and Animal Welfare), 2023). This transition has been definitively stated by the European Resolution P9_TA(2021)0295, which answering the European Citizens’ Initiative “End the Cage Age,” asks the European Commission to ban any cage system for farmed animals within the European Union, laying hens included, by 2027.

However, cage-free systems have been associated with increased hygiene risks and loss of efficiency for egg production, besides animal welfare concerns other than restricting some species-specific behaviors (Hemsworth, 2021; Michel et al., 2022). As for egg production and farm efficiency, the main concern is about the laying of variable proportions of eggs on the floor rather than in the nests, which increases discarded eggs, and thus decreases profitability, besides increasing the farmers’ labor due to the hand collection of the eggs from the floor (Oliveira et al., 2016; Villanueva et al., 2017). Compared to cage-systems, increased mortality has been reported in cage-free systems (Hemsworth, 2021), even if improvements in projecting and experience in managing these systems have been recently claimed to play a positive role (Schuck-Paim et al., 2021). As for animal welfare concerns, the free movement of hens is also associated with piling and smothering on the floor and at partitions and corners inside the aviary, besides in the nests due to overcrowding at laying time (Kruschwitz et al., 2008; Hemsworth, 2021). These events cause stress to laying hens, possibly contributing with other factors to the occurrence of feather pecking, which can end in severe injuries till cannibalisms under commercial conditions (Hemsworth and Edwards, 2020; Michel et al., 2022). Additionally, navigation in the complex environment of cage-free systems can result in failures and collisions. While these latter can cause various bone damages, such as keel deviations and fractures, depending on other predisposing factors (Toscano et al., 2020), undoubtedly, a high rate of navigation failures can be interpreted as a limitation of movement even in the open space of cage-free systems and, thus, as a welfare concern. Differences in navigation ability and space use in an aviary system can depend on genotype, aviary design, the management of pullets at housing, and last but not least, the early experience of pullets (Purdum et al., 2020; Pufall et al., 2021; Du et al., 2022). Several studies are available on animal distribution and space use during the laying period (Ali et al., 2016, 2020; MacLachlan et al., 2020; Purdum et al., 2020), while few of them specifically focused on the first weeks after housing of pullets in the new barn (Colson et al., 2008; Zheng et al., 2019).

In fact, hens must navigate easily between the different levels of the aviary to reach feed and water for their health and body development; they must distribute homogeneously in all levels of the aviary for full space use; they must quickly identify the different areas of the aviary for the full and safe expression of all their specie-specific behaviors and for functional use of the different areas to prevent floor egg laying. In this regard, perches allow birds to exercise and use the vertical space within the housing system; they allow for vigilance and roosting at night (Schrader and Müller, 2009; Brendler et al., 2014); they contribute to muscle development and bone mineralization, which can reduce bone damages, besides improving feather plumage, and foot and nail health (Hester, 2014; Hemsworth and Edwards, 2020).

Thus, the present study was designed to test the hypothesis that increased availability of perches could favor the adaptation of pullets in the new aviary system for laying and their navigation ability and it could reduce competition among animals, and thus stress, for perching sites. To this purpose, the nightly and daily distribution of hens during the first 4 wk after housing at 17 wk of age were compared in 2 genotypes (White and Brown) housed in pens of an experimental aviary enriched or not with additional perches, besides those included in the structure of the aviary. Moreover, the navigation ability of hens, as for the landings from the aviary to the floor and the take-offs from the floor to the aviary, was compared at 17 and 20 wk of age.

## MATERIALS AND METHODS

### Ethics Statement

The Ethical Committee for Animal Experimentation of the University of Padova approved the study (project 28/2020; Prot. n. 204398) that followed the principles of the EU Directive 2010/63/EU.

### Experimental Facilities, Animals, and Experimental Arrangement

**Figure 1:**
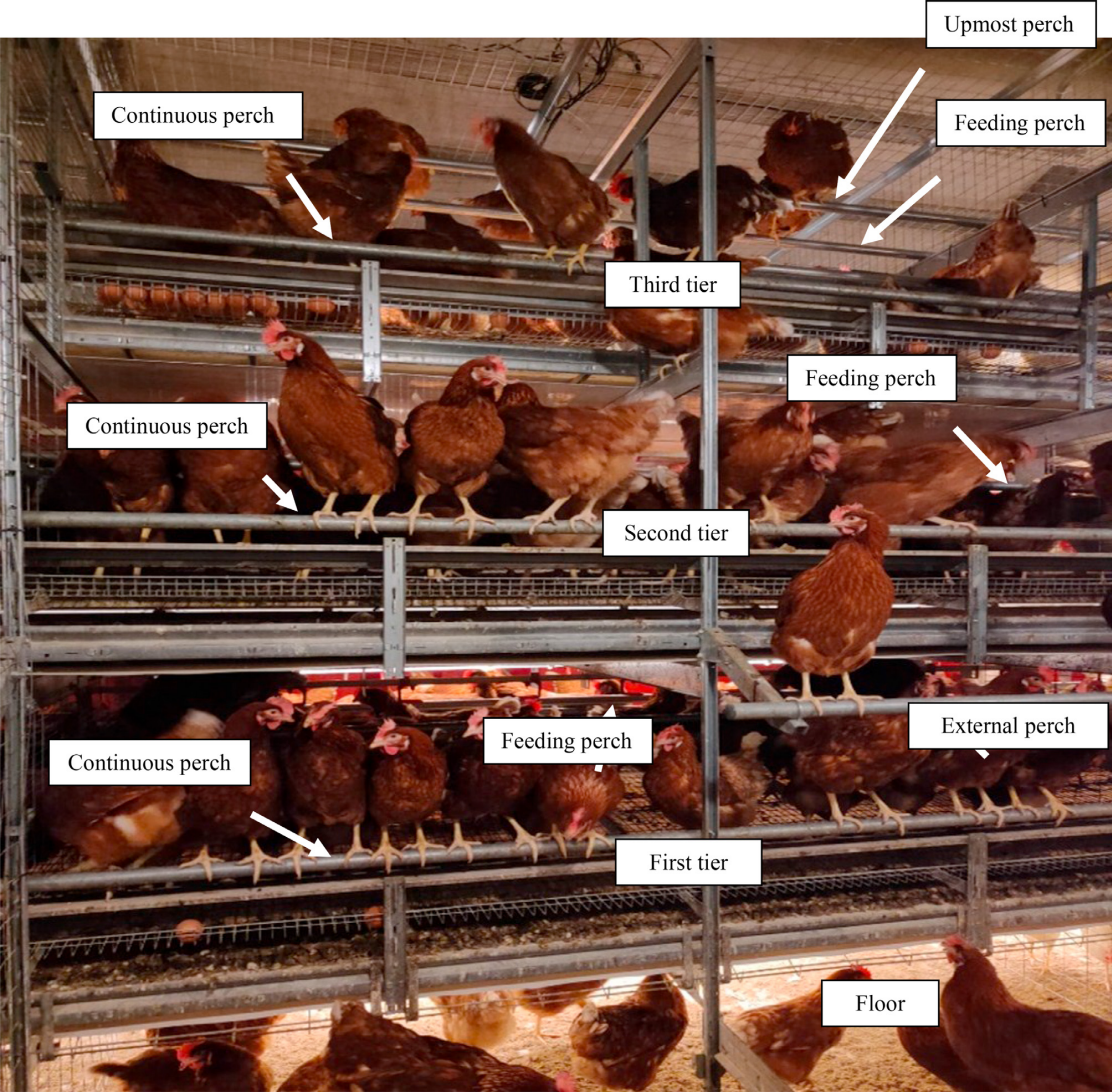
Front of the aviary with identification of tiers and perches.

**Figure 2:**
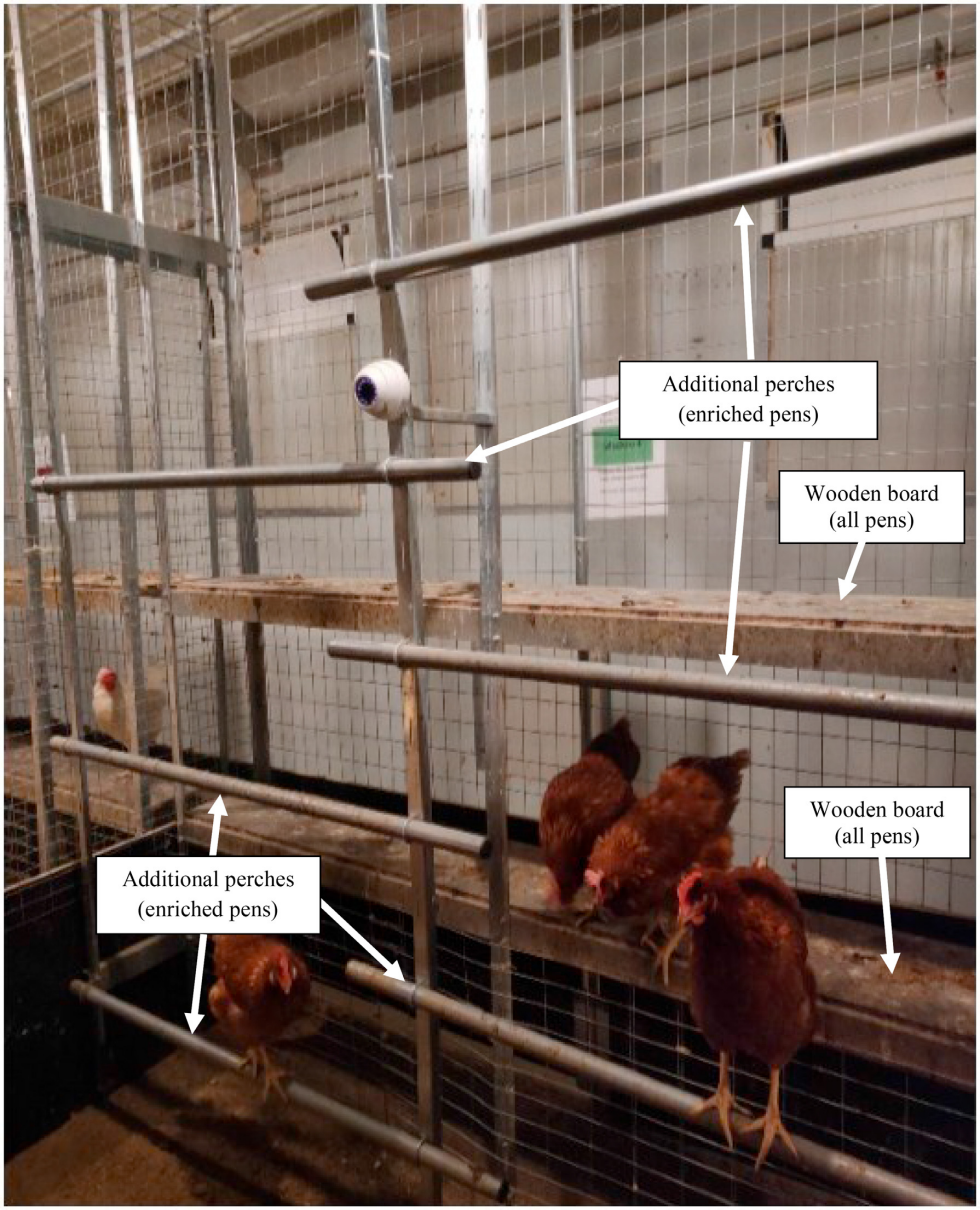
Outer wall in front of the aviary equipped with 2 wooden boards (all pens) and 6 additional perches (only in the 4 enriched pens).

Hens were housed in a stable at the Experimental Farm of the University of Padova (Legnaro, Padova, Italy) equipped with controlled lighting and heating systems, a cooling system, and forced ventilation. The experimental aviary was specifically set up for the present study. The aviary consisted of 3 tiers. The first 2 tiers were equipped with collective nests (1 nest per 60 hens) closed by a series of plastic curtains (5 curtains of 18 cm per nest separated by 5 cm), continuous perches over the tiers (both tiers), feeding perches (both tiers), and external perches (only the second tiers; length 120 cm), nipple drinkers, and automatic feeders (Figure 1). The third tier had only automatic feeders, continuous perches, feeding perches, and uppermost perches along the whole length of the pen (Figure 1). The whole experimental aviary system was 2.25 m wide × 19.20 m long × 3.00 m high. A corridor was available (3.30 m wide) adjacent to the aviary, so the floor space was 5.55 m wide × 19.20 m long. The aviary was then divided into 8 pens, each with a length of 2.40 m. On the outer net wall of the corridor of the aviary, all pens had 2 wooden boards (0.30 m wide × 2.40 m long) at the height of 0.86 m and 1.66 m (Figure 2). Moreover, in 4 pens (enriched pens), the outer net walls were equipped with 6 additional perches, each 1.20 m long, alternated, and placed at different heights (0.30 m, 0.90 m, and 1.50 m on the left side of the wall; 0.60, 1.20 m, and 1.80 m on the right side of the wall) (Figure 2), corresponding to 3.2 cm perches/hen. Starting from the ground, the first perch was positioned at 0.30 m to facilitate the hen use and access to perches especially at the housing time, that is, at the arrival in the farm; all the other perches were positioned at a height equal or higher than 0.60 m based on the hen preference for high perches for night-time roosting (EFSA AHAW Panel (EFSA Panel on Animal Health and Animal Welfare), 2015). The total available linear space was 15.2 cm and 18.4 cm of perches per hen in not enriched and enriched pens, respectively.

A real-time video recording system was used with a total of 48 cameras (Infrared mini-dome bullet 4 mp; resolution 1,080 p) (HAC-HDW1220MP, Zhejiang Dahua Technology Co., Ltd., Hangzhou, China) and 2 full HD video-recorders (NVR2116HS-4KS2, Zhejiang Dahua Technology Co., Ltd., Hangzhou, China). The cameras were located to record hens on the ground and the equipment of the outer wall (1 camera per pen hanged at about 3 m of height); hens on the first and second tier of the aviary (1 camera per pen hanged at about 1.50 m of height on the outer wall); hens on the third tier of the aviary (1 camera per pen hanged at about 2.80 m of height on the outer wall); hens in the nests (1 camera per nest hanged at the inside left corner of the nest); and hens on the third tier (1 camera per pen hanged over the third tier). The system was set up to get and store 24-h videos once per week using all the cameras.

A total of 1,800 hens, 900 Hy-line Brown (Hy-Line International, West Des Moines, IA) and 900 Lohmann White-LSL (Lohmann Tierzucht GmbH, Cuxhaven, Germany), aged 17 wk, were delivered by an authorized truck from the same commercial farm to the experimental farm. On arrival, hens were randomly allocated in the 8 pens of the aviary (225 hens per pen; 9 hens/m^2^ available surface). Four experimental groups, with 2 pens per group, were thus obtained according to a bifactorial arrangement with 2 genotypes (Brown vs. White hens) and 2 types of pens (not enriched vs. enriched with additional perches).

During the period of the trial (17–20 wk of age), all hens were fed 2 commercial diets. Minimum and maximum temperatures inside the barn averaged 20.1°C ± 0.9°C and 23.9°C ± 0.5°C, respectively, with average minimum and maximum relative humidity at 68.6 ± 6.9% and 85.1 ± 4.8%, respectively.

Hens arrived in the experimental farm in July. In the origin farm, they had been kept under a natural photoperiod. Thus, in agreement with the technician of the company supplying the hens, 16 h of light were provided during the first week after their arrival (17 wk of age), which increased to 16.5 h of light in the second week (18 wk of age) to continue with the photostimulation. Dimmers were used for sunset and sunrise. The sunrise was set at 7 min for the lamps inside the aviary followed by 5 min for the lamps in the corridors; the sunset was 10 min in the corridors, followed by 17 min inside the aviary. The nests were always opened with free access for hens during the 4 wk of recordings.

A programmable logic controller (Officine Facco & C. Spa, Campo San Martino, Padova, Italy) managed feeding, drinking, lighting, and ventilation inside the system.

### Animal-Based Recordings

From the arrival of animals until 20 wk of age, once per week, 50 hens per pen were randomly taken to measure their live weight; to assess the feather condition of neck, head, back, and cloaca zones (score 0: no or limited damage; 1: moderate damage with areas <5 cm in diameter without feathers; 2: skin with areas ≥5 cm in diameter without feathers) (Van Niekerk et al., 2012); foot pad lesions (0: no lesions; 1: few lesions as hyperkeratosis or small injuries; 2: many lesions with swelling of the foot dorsally visible known as bumble foot) (Van Niekerk et al., 2012); keel bone damage (0: no damage; 1: keel bone deviation) (Welfare Quality Assessment Protocol for Poultry, 2009).

### Distribution and Space Use of Hens in the Aviary

To evaluate the distribution and space use of hens in the aviary during the night, videos recorded once per week were used to score the position of the hens 120 min after switching off the light, at 17, 18, 19, and 20 wk of age. In detail, videos were scanned to obtain the number of hens per pen on the floor, on the wooden boards, on the additional perches (in the enriched pens), in the nests of the 2 tiers of the aviary, on the aviary external and feeding perches of the 3 tiers.

Finally, to evaluate the space use during the day, 2 trained operators recorded the distribution of hens in the aviary by direct observation once per week at 18, 19, and 20 wk of age within each pen at 11.00 h, 1 h after the automatic distribution of feed. Direct observations were added to nightly video-recordings because cameras did not get all hens on the wire net of the tiers of the different levels. Recordings started at 18 wk of age to wait hens were more familiar with the presence of operators in the barns. To minimize potential disruptions to the usual behavior of hens, observations were made after all the other daily operations; observers were the same people in charge of all other daily recordings in the farm; observers did not talk between them, quietly moved along the external corridor, and got the measures 5 min after stationing nearby the pen. In detail, the number of hens in the different areas of the aviary (floor; wire mesh of the first, second, and third tier; feeding perches and external perches of the first, second, and third tier; upper perches of the third tier and nests), besides the number of hens on the wooden boards and additional perches (when present) of the outer wall were scored.

### Navigation Activity and Ability of Hens

To measure the navigation ability of hens over time, the 24-h videos recorded at 17 and 20 wk of age were used. The total number of landings from any part of the aviary system to the floor and the wooden boards and additional perches of the outer walls, and the total number of take-offs from the floor to any part of the aviary system (including the outer wall) were scored during the first 10 min of every hour of light (from 5.00 to 21.00). Moreover, when the displacement (flight or jump), whatever the direction, resulted in an uncontrolled movement (collision with any part of the aviary or with other animals), it was considered a failed displacement; otherwise, if the hens reached the areas without collision, the displacement was considered successful (Stratmann et al., 2019).

### Statistical Analysis

Data of nightly and daily distribution of hens in the aviary at different ages were analyzed by a generalized linear mixed model using the PROC GLIMMIX of SAS (SAS Institute, 2013) with the week of age, enrichment with additional perches, and genotypes as main effects with interactions and the pen as a random effect. The same model was used for the statistical analyses of data regarding the number of landings and take-offs and the rate of successful and failed displacements, including as the main effect also the time-interval of observation (i.e., early, midday, afternoon, and late, corresponding to the intervals 5.00–8.00 h, 9.00–12.00 h, 13.00–16.00 h, and 17.00–21.00 h). The least square means were compared using Tukey's *t* test. Differences between the means with *P* ≤ 0.05 were considered statistically significant.

Results for the main effects are given in the tables of the manuscript. In Supplementary materials, Table S1 reports the data for live weight and lesions; Tables S2a to S5f report all results for the interactions among the main experimental factors as for nightly and daily distribution of hens, landings, and take-offs.

To identify the risk factors related to failed landings of pullets at housing in the aviary system for laying, the effects of age, genotype, time-interval of observation, and flight distance (i.e., short from the first tier; medium from the second tier; long from the third tier), and the enrichment with additional perches on the outer wall were evaluated by univariate and multivariate logistic regression analysis using the PROC LOGISTIC of SAS. Initially, factors were screened for multicollinearity (correlation coefficient |*r*| < 0.7), and univariate analysis was performed for each independent factor (Table S6). Then, variables that showed a *P* < 0.05 in the univariate analysis were included in a multivariate logistic regression analysis and the risk factors were identified through a stepwise forward selection based on *P* < 0.05. The regression coefficients were expressed as odds ratio (**OR**) with a 95% confidence interval (**CI**).

## RESULTS AND DISCUSSION

### Distribution of Hens and Space Use

Different studies have shown how the complexity of the environment extends the bird behavioral repertoire (Sosnówka-Czajka et al., 2021). On the other hand, the response of pullets and their ability to adapt, use the space, and freely navigate in a complex system at the time of housing in the new barn depend on several factors, out of which previous experience, available equipment, and genotype play a key role (Ali et al., 2020; Sulimova et al., 2020).

In the present study, we scored pullet distribution in the experimental aviary as an indicator of space use and hen preferences during the first 4 wk upon arrival in the new farm (Tables 1 and 2). These first weeks are crucial for welfare and later performance, because at this time young hens have not still reached their full somatic development, and they need to access quickly and freely to feed and water. Additionally, at housing in the new barn, hens must get familiar with the different equipment and areas of the aviary, assigning them a specific functional role (Wolc et al., 2021).

**Table 1:** Effect of age, enrichment with additional perches, and genotype on the rate of hens (% of hens per pen) (means ± SD) on the floor, on the equipment of the aviary and of the outer wall during the night.

	Week of age (A)				Additional perches (P)		Genotype (G)		*P* value		
Variables	17	18	19	20	No	Yes	White	Brown	A	P	G
Observations (*n*)	8	8	8	8	16	16	16	16			
Floor (%)	8.36^B^ ± 8.70	2.66^AB^ ± 4.93	1.49^A^ ± 1.81	1.06^A^ ± 1.84	3.96 ± 6.52	2.83 ± 5.18	0.15 ± 0.62	6.63 ± 6.97	<0.001	0.140	<0.001
Aviary											
First tier											
Nests (%)	2.01^b^ ± 2.15	1.78^ab^ ± 1.89	1.61^ab^ ± 2.12	1.54^a^ ± 1.84	1.00 ± 1.48	2.47 ± 2.18	0.86 ± 1.57	2.61 ± 2.01	<0.05	<0.001	<0.001
Continuous perch (%)	1.33 ± 1.50	1.22 ± 1.56	1.11 ± 1.35	1.45 ± 1.89	1.34 ± 1.74	1.22 ± 1.30	0.08 ± 0.24	2.48 ± 1.27	0.896	0.372	<0.001
Feeding perch (%)	1.56 ± 1.78	1.78 ± 1.93	1.50 ± 1.73	1.48 ± 1.31	1.66 ± 1.68	1.51 ± 1.66	0.00 ± 0.00	2.97 ± 0.90	0.343	0.431	<0.001
Second tier											
Nests (%)	0.78^b^ ± 1.43	1.09^b^ ± 1.23	0.39^a^ ± 0.59	0.34^a^ ± 0.52	0.56 ± 0.99	0.74 ± 1.12	0.53 ± 1.13	0.77 ± 0.97	<0.05	0.828	0.828
External perch (%)	0.44 ± 0.63	0.11 ± 0.21	0.39 ± 0.60	0.78 ± 1.08	0.39 ± 0.63	0.47 ± 0.80	0.03 ± 0.11	0.83 ± 0.83	0.153	0.863	<0.01
Continuous perch (%)	2.00^a^ ± 1.82	2.67^ab^ ± 3.04	2.78^ab^ ± 2.92	4.12^b^ ± 3.12	2.92 ± 2.75	2.86 ± 2.84	0.67 ± 0.69	5.12 ± 2.15	<0.05	0.530	<0.001
Feeding perch (%)	0.25 ± 0.35	0.44 ± 0.67	0.45 ± 0.34	0.89 ± 1.14	0.36 ± 0.33	0.68 ± 0.96	0.15 ± 0.28	0.86 ± 0.83	0.056	0.196	<0.001
Third tier											
Continuous perch (%)	6.42^A^ ± 0.63	7.96^B^ ± 1.65	8.91^B^ ± 1.09	8.47^B^ ± 1.44	8.53 ± 1.32	7.41 ± 1.57	8.44 ± 1.53	7.56 ± 1.45	<0.01	<0.01	<0.05
Feeding perch (%)	0.32 ± 0.22	0.19 ± 0.35	1.02 ± 0.84	1.17 ± 1.24	0.77 ± 0.76	0.62 ± 0.99	1.03 ± 1.17	0.42 ± 0.38	0.065	0.636	0.186
Upmost perch (%)	3.05^a^ ± 2.42	4.38^ab^ ± 1.96	4.83^b^ ± 1.40	4.74^b^ ± 1.44	4.30 ± 1.96	4.24 ± 1.86	5.79 ± 0.96	3.03 ± 1.48	<0.05	0.966	<0.001
Outer wall											
Additional perches (%)	1.83^B^ ± 1.02	1.91^B^ ± 0.98	1.77^B^ ± 0.92	1.39^A^ ± 0.83	-	3.45 ± 1.90	2.47 ± 0.69	0.98 ± 0.48	<0.001	-	<0.001
Wooden boards (%)	2.47 ± 2.41	2.14 ± 2.00	2.32 ± 2.16	2.18 ± 2.18	3.43 ± 2.21	1.13 ± 1.41	2.21 ± 2.09	2.34 ± 2.27	0.056	<0.001	<0.001

Data were collected once per week at 2 h after switching off the lights of the barn from video recordings; cameras did not get hens on the wire nets of the tiers of the different levels.

a,b Means with different superscript letters are different (*P* < 0.05).

A,B Means with different superscript letters are different (*P* < 0.01).

**Table 2:** Effect of age, enrichment with additional perches, and genotype on the rate of hens (% of hens per pen) on the different levels of the aviary (means ± SD) during the day.

	Week of age (A)			Additional perches (P)		Genotype (G)		*P* value		
Variables	18	19	20	No	Yes	White	Brown	A	P	G
Observations (*n*)	8	8	8	12	12	12	12			
Floor (%)	22.2 ± 2.76	19.4 ± 3.83	19.8 ± 5.54	21.6 ± 5.04	19.4 ± 2.99	18.3 ± 4.19	22.6 ± 3.12	0.141	0.076	<0.05
Aviary										
First tier (%)	27.6 ± 8.64	25.9 ± 9.74	25.4 ± 7.74	27.0 ± 6.89	25.6 ± 9.97	18.8 ± 3.25	33.8 ± 3.58	0.407	0.267	<0.001
Continuous perch (%)	5.16 ± 1.46	5.27 ± 1.56	5.02 ± 1.82	5.62 ± 1.10	4.69 ± 1.83	4.03 ± 1.31	6.27 ± 0.78	0.833	<0.05	<0.001
Wire mesh (%)	14.5 ± 3.99	13.7 ± 4.81	13.8 ± 3.52	14.5 ± 4.06	13.6 ± 4.00	10.5 ± 1.74	17.5 ± 1.73	0.665	0.273	<0.001
Feeding perch (%)	1.46 ± 1.72	0.91 ± 1.09	1.25 ± 1.28	1.09 ± 1.21	1.33 ± 1.51	0.09 ± 0.20	2.34 ± 1.00	0.313	0.423	<0.001
Nests (%)	6.42 ± 3.01	5.98 ± 2.79	5.26 ± 1.62	5.82 ± 1.54	5.96 ± 3.24	4.09 ± 0.94	7.69 ± 2.22	0.364	0.839	<0.001
Second tier (%)	22.6 ± 5.01	22.6 ± 3.59	22.1 ± 5.88	22.5 ± 3.89	22.3 ± 5.58	19.1 ± 3.83	25.8 ± 2.61	0.932	0.850	<0.001
Continuous + external perch (%)	4.77 ± 1.08	6.12 ± 1.83	5.25 ± 1.09	5.59 ± 1.74	5.18 ± 1.10	5.18 ± 1.72	5.59 ± 1.12	0.160	0.468	0.464
Wire mesh (%)	12.0 ± 3.01	12.1 ± 1.71	12.5 ± 4.11	12.3 ± 2.49	12.1 ± 3.51	10.2 ± 2.01	14.2 ± 2.35	0.789	0.771	<0.01
Feeding perch (%)	0.58 ± 0.84	0.29 ± 0.36	0.63 ± 0.52	0.54 ± 0.59	0.45 ± 0.63	0.16 ± 0.31	0.84 ± 0.63	0.266	0.898	<0.001
Nests (%)	5.21^b^ ± 1.99	4.10^ab^ ± 0.85	3.73^a^ ± 1.37	4.11 ± 1.24	4.58 ± 1.83	3.54 ± 0.87	5.15 ± 1.69	<0.05	0.219	<0.01
Third tier (%)	22.4 ± 8.32	24.4 ± 7.25	25.8 ± 14.0	24.6 ± 9.93	23.7 ± 10.4	32.8 ± 5.74	15.6 ± 3.57	0.202	0.928	<0.001
Continuous perch (%)	1.74^A^ ± 1.09	2.95^B^ ± 0.75	1.59^A^ ± 0.71	2.42 ± 0.57	1.77 ± 1.30	2.55 ± 0.80	1.64 ± 1.07	<0.001	<0.05	<0.01
Wire mesh (%)	16.8 ± 4.85	15.8 ± 3.48	16.6 ± 7.57	16.4 ± 5.25	16.4 ± 5.66	20.4 ± 3.75	12.4 ± 3.24	0.804	0.901	<0.001
Feeding perch (%)	0.47 ± 0.49	1.00 ± 0.89	1.28 ± 1.56	0.92 ± 1.06	0.91 ± 1.15	1.59 ± 1.19	0.25 ± 0.26	0.136	0.770	<0.01
Upmost perch (%)	3.35^a^ ± 2.86	4.60^ab^ ± 3.74	6.30^b^ ± 5.51	4.89 ± 4.60	4.61 ± 3.92	8.22 ± 3.04	1.28 ± 1.01	<0.05	0.926	<0.001
Outer wall										
Additional perches (%)	2.43 ± 2.21	2.97 ± 2.06	1.78 ± 1.24	0.00	4.78 ± 3.54	3.88 ± 1.77	0.91 ± 0.29	0.238	-	<0.01
Wooden boards (%)	2.87 ± 2.77	4.73 ± 4.08	5.12 ± 4.09	4.25 ± 4.02	4.24 ± 3.47	7.17 ± 2.89	1.31 ± 1.10	0.152	0.993	<0.001

Data were collected once per week at 11:00 by direct observation.

a,b Means with different superscript letters are different (*P* < 0.05).

A,B Means with different superscript letters are different (*P* < 0.01).

Under our conditions, after housing, the main differences at night were observed in the rate of hens on the floor, which decreased from 8.36% of hens per pen in the first week to 1.49 and 1.06% at 19 and 20 wk of age (*P* < 0.001) (Table 1). In other words, within 2 wk after housing, all animals used the aviary and the equipment of the outer wall for resting during the night. A short time for adaptation was also observed in a previous study using Brown hens (Pillan et al., 2020), where the number of hens on the floor after turning off the light decreased within 15 d after housing. Then, in the present study, changes across the 4 wk in the use of the additional perches of the outer wall were in a quite narrow range, showing the highest value at 18 wk of age and the lowest one at 20 wk of age (1.91 vs. 1.39% of hens per pen; *P* < 0.001) (Table 1). On the other hand, after housing, the rate of animals recovering in the nests at night significantly decreased, both at the first (2.01 vs. 1.54% hens per pen at 17 vs. 20 wk of age; *P* < 0.05) and at the second tier (0.78 and 1.09% at 17 and 18 wk vs. 0.39 and 0.34% at 19 and 20 wk of age; *P* < 0.05). This reduction should be positively considered as nests have to be used as places for laying eggs and not for resting. Then, hens soon used the perches of the first tier, but they took 3 wk to increase the use of the continuous perches of the second tier (from 2.00 to 4.12% of hens per pen from 17 to 20 wk of age; *P* < 0.05) and of the upmost perches (from 3.05 to 4.74%; *P* < 0.05) (Table 1). Moreover, only 1 wk was necessary to reach the continuous perches (6.42% hens per pen at 17 wk of age vs. 7.96, 8.91, and 8.47 at 18, 19, and 20 wk, respectively; *P* < 0.01) of the third tier.

Daily observations of hen distribution in the aviary (Table 2) confirmed results recorded during the night, as for the enhanced ability of hens to reach the upmost levels of the experimental aviary as age increased. In fact, from 18 to 20 wk of age, the rate of hens on the upmost perches of the third level increased (from 3.35 to 6.30%; *P* < 0.05), whereas the rate of hens in the nests of the second tiers decreased (from 5.21 to 3.73%; *P* < 0.05) (Table 2).

Indeed, early adaptation of hens to the rearing environments for laying is crucial for the development of species-appropriate behavior and optimal physical growth (Janczak and Riber, 2015; Pullin et al., 2020). The inability of young hens to navigate the 3-dimensional space in the new barn after housing would lead to different negative issues, such as fear, which can cause stress and choking, besides difficulty in locating resources (Janczak and Riber, 2015). On the other hand, pullets trained during growth by suitable resources (e.g., elevated levels or ramps) show earlier use of aviary levels and improved navigation ability (as for length and success of flights and jumps), besides higher use of nests during laying (Colson et al., 2008). According to Tahamtani et al. (2014), trained pullets are better at solving spatial problems due to their improved working memory. Nevertheless, under our conditions, despite coming from a farm without any equipment for training to vertical movements, hens got the first and second tiers of the aviary, where both feed and water were available, immediately after housing in the new barn; they left the floor to rest on the aviary at night in 1 wk; however, they needed more time to reach the upper part of the aviary (third tier).

Under our conditions, the contribution of additional perches on the outer wall of the pen to the adaptation of pullets to the experimental aviary and to the balanced distribution of animals was not relevant. The presence of additional perches obviously decreased the rate of hens standing on the wooden boards of the outer wall, because hens used both perches and boards; at the same time, it increased the rate of hens in the nests after switching off the lights, especially at the first tier (1.00 vs. 2.47%; *P* < 0.001) (Table 1). This result has not a clear explanation: it could be argued that the presence of the additional perches stimulated hen activity and exploration of the aviary at housing for which hens soon reached the nests of the aviary compared to what happened in the standard pens. On the other hand, at night, the presence of additional perches decreased the rate of hens on the continuous perches of the third tiers (8.53 vs. 7.41%; *P* < 0.01), which likely depended on the higher availability of perches where roosting at night in the enriched pens compared to the standard ones. These results were confirmed by the daily observations: the rates of hens on the continuous perches of the first (4.69 vs. 5.62%; *P* < 0.05) and the third tiers (1.77 vs. 2.42%; *P* < 0.05) were lower in pens with additional perches compared to standard pens (Table 2).

As for genotype, hens of different genotypes are known to behave and use space differently (Schütz and Jensen, 2001; Schütz et al., 2001; Ali et al., 2016; Purdum et al., 2020), as found in the present study. At night, we recorded a lower rate of hens on the floor (0.15 vs. 6.63%) and a higher rate of hens on the additional perches (2.47 vs. 0.98%) in White compared to Brown hens (*P* < 0.001) (Table 1). These results show a faster adaptation of White hens to the experimental aviary, besides a preference of these hens for the upmost levels of the aviary. White hens also used nests of the first tier for resting during the night to a lower extent compared to Brown hens (0.86 vs. 2.61%; *P* < 0.001) (Table 1). Then, at night, White hens did not use perches of the first or the second tiers, differently from Brown hens, and crowded the third tier for sleeping on the external and upper most perches (*P* < 0.001) (Table 1). Moreover, at night, under our conditions, despite it was not possible to objectively record the number of animals on the wire nets, White hens were occasionally found on the first and the second tiers, whereas they crowded the third level.

Indeed, in the first week after housing (17 wk of age), the rate of hens observed at night in the nests of the first tier was higher for White compared to Brown hens (2.60 and 1.41% of hens per pen, respectively); then, an opposite behavior was observed during the following weeks (0.66 and 2.91% in White and Brown hens at 18 wk of age; 0.04 and 3.18% at 19 wk; 0.15 and 2.92% at 20 wk) (probability of the interaction genotype  × week of age, *P* < 0.001) (Table S2a). The same differences were also recorded for the rate of hens in the nests of the second tier (probability of the interaction genotype  × week of age, *P* < 0.01) (Table S2a). Then, the rate of hens on the wooden boards of the outer wall at night was lower in White compared to Brown hens only at 17 wk of age (1.93 vs. 3.02%) without differences between genotypes in the following weeks (2.12 and 2.16% in White and Brown hens at 18 weeks of age; 2.48 and 2.15% at 19 wk; 2.33 and 2.03% at 20 wk) (probability of the interaction genotype  × week of age, *P* < 0.001) (Table S2a).

The differences between White and Brown hens in the space use at night were confirmed by daily observations (Table 2). The rates of White hens on the third tier (32.8 vs. 15.6%; *P* < 0.001), the additional perches (3.88 vs. 0.91%; *P* < 0.01), and the wooden boards (7.17 vs. 1.31%; *P* < 0.001) of the enriched wall were higher compared to Brown hens. Conversely, the rates of the White compared to Brown hens were lower on the floor (18.3 vs. 22.6%; *P* < 0.05), the first tiers (18.8 vs. 33.8%; *P* < 0.001), and the second tiers (19.1 vs. 25.8%; *P* < 0.001). The lower rate of White hens on the feeding perches of the first tier, especially, and the second tier can be likely associated with a lower feeding activity compared with Brown hens (*P* < 0.001). An opposite result was observed at the feeding perches of the third tiers (1.59 vs. 0.25% in White hens vs. Brown hens; *P* < 0.01) where White hens stayed for roosting. Finally, at the daily observations, a lower rate of White compared to Brown hens was found in nests both at the first (4.09 vs. 7.69%; *P* < 0.001) and at the second tiers (3.54 vs. 5.15%; *P* < 0.01) (Table 2). When age increased, the difference between the 2 genotypes in the rate of hens observed on the third tiers increased from 18 and 19 wk of age (+14.4 and +12.4 percentage points in White vs. Brown hens) to 20 wk of age (+24.8 percentage points in White vs. Brown hens) (probability of the interaction genotype  × week of age, *P* < 0.01) (Table S3a).

Previous studies in laying hens also found that the preference for different levels of the aviary or perches depended on genotype, besides the daily observation time (Odén et al., 2002; Brendler et al., 2014; Brendler and Schrader, 2016; Campbell et al., 2016a). On the whole, these results confirm the preference we found in young White hens for the upper levels of the aviary both at daily and nightly observations compared to Brown hens. In detail, during the day, Ali et al. (2019) reported that White hens stayed more on ledges and perches, whereas Brown hens were more on the wire mesh of the aviary. At night, Ali et al. (2016) found that White hens stayed more on the upper levels of the aviary compared to Brown hens, whereas an opposite trend was recorded in the morning. Campbell et al. (2016b) observed more White hens on perches at night than during the day (45.1 vs. 25.5% of hens per pen, respectively), which is consistent with the observations of Silversides et al. (2012). Giersberg et al. (2019) reported that during the day dual-purpose hens (Lohmann dual) used more the lower perches, whereas conventional layer hens the higher perches. When age of laying hens increased, Wolc et al. (2021) found that perching tended to increase, and the proportion of eggs laid on the floor tended to decrease, which means that these are learned behaviors. Interestingly, the same authors also found that, genetically, there was a positive correlation between use of perches and use of nests. In fact, Purdum et al. (2020) showed that perch use was affected by the interactions between different factors, that is, genotype, hour of the day, and age of hens during the laying periods.

In our study, some significant interactions were recorded between genotype and enrichment with additional perches. In detail, the highest rate of hens in the nests of the first tier was recorded for Brown hens in enriched pens (not enriched pens: 0.69 and 1.30% for White and Brown hens; enriched pens: 1.04 and 3.91% for White and Brown hens; *P* < 0.01) (Table S2b), which corresponded to the lowest rate of hens on the wooden boards (not enriched pens: 2.65 and 4.21% for White and Brown hens; enriched pens: 1.78 and 0.47% for White and Brown hens) (probability of the interaction genotype × enrichment, *P* < 0.01) (Table S2b). As for the daily observations, the highest difference between genotypes in the rate of hens on the first on the first and the second tiers was recorded in the enriched pens (first tier, not enriched pens: 20.8 and 33.2% for White and Brown hens; enriched pens: 16.6 and 34.5% for White and Brown hens; *P* < 0.10) (second tier, of not enriched pens: 20.7 and 24.4% for White and Brown hens; enriched pens: 17.5 and 27.2% for White and Brown hens; *P* < 0.01) (Table S3b). In other words, the enrichment with additional perches further reduced the use of the first and the second tiers of the aviary in White compared to Brown hens.

Thus, despite the present study had some limitations (low number of units per experimental group, i.e., 2 pens; lower number of animals per group compared to field conditions, i.e., 225 hens per pen), the experimental set up permitted us to get information under controlled conditions about space use by hens at housing in the new barn that would be difficult to get in the field. This information importantly included differences in hen preferences and aptitudes according to genotypes and interactions of genotypes with the other main factors, which have to be taken into account when designing cage-free systems to optimize animal welfare.

### Navigation Activity and Ability

At housing in the new barn, the stress of young hens could be accentuated by difficulties in navigating in an environment with different distribution of resources, different angles, and distances, as well as different light management, compared with the pullet phase. These difficulties might result in falls and collisions with equipment or other animals (Stratmann et al., 2019), which can be associated with movement restriction and fear, besides possible lesions.

Based on the data collected in the present trial, the preliminary univariate analysis identified week of age, flight distance, equipment of the outer wall (with or without additional perches), genotype, and observation time as the potential influencing factors (*P* < 0.05) for failed landing in laying hens, while the enrichment of the outer wall with additional perches played a minor role (*P* = 0.09) (Table S6). Thus, the forward selection of the multivariate logistic regression analysis extracted almost all the same significant factors and calculated the odds ratio for the occurrence of failed landings (Table 3). We did not run the same analysis for take-off events due to the “quasi-complete separation of data points,” which was associated with the low number of failed displacements recorded across the observations.

**Table 3:** Factors influencing failed landings in laying hens from 17 to 20 wk of age and extracted by forward selection in a multivariate logistic regression analysis.

				95% CI		
Variable	Estimate	SE	Odds ratio	Lower	Upper	*P* value
Intercept	−2.77	0.18				<0.001
Week of age						
20 (Ref)	-	-	-	-	-	
17	0.32	0.11	1.90	1.21	2.97	<0.01
Flight distance from aviary to floor						
Medium (Ref)	-	-	-	-	-	
Short	−2.34	0.22	0.17	0.09	0.31	<0.001
Long	2.89	0.19	31.2	18.9	51.3	<0.001
Genotype						
White (Ref)	-	-	-	-	-	-
Brown	0.98	0.12	7.22	4.46	11.7	<0.001
Observation time^1^						
Afternoon (Ref)	-	-	-	-	-	-
Midday	0.61	0.22	0.98	0.56	1.70	<0.01
Early	−2.40	0.46	0.05	0.01	0.17	<0.001
Late	1.16	0.21	1.70	1.03	2.82	<0.001

SE = standard error; CI = confidence interval; Ref = reference.

1 Early, observations from 5:00 to 8:00; Midday: observations from 9:00 to 12:00; Afternoon: observations from 13:00 to 16:00; Late: observations from 17:00 to 21:00.

The average number of landings per observation time increased by 2.29 times (from 31.2 to 71.4; *P* < 0.001), and the rate of successful landings increased from 89.5% at 17 wk to 93.9% at 20 wk of age (*P* < 0.01) (Table 4). The logistic regression analysis showed significantly higher odds (OR: 1.90; *P* < 0.001) of experiencing failed landings at 17 wk compared to 20 wk (Table 3). Take-offs from the floor to whatever level of the aviary or the enriched wall increased by 3.19 times (from 20.1 to 64.2; *P* < 0.001) and the rate of successful landings was not affected (Table 5).

**Table 4:** Effect of age, enrichment with additional perches, genotype, and observation time on landings (starting point the aviary) in a cage-free aviary system (means ± SD).

	Week of age (A)		Additional perches (P)		Genotype (G)		Observation time^1^ (T)				*P* value			
Variables	17	20	No	Yes	W	B	Early	Midday	Afternoon	Late	A	P	G	T
Observations (n)	32	32	32	32	32	32	16	16	16	16				
Landings per observation time	31.2 ± 24.8	71.4 ± 42.5	55.3 ± 40.8	47.3 ± 39.5	80.7 ± 35.4	21.9 ± 14.7	62.2^b^ ± 53.8	48.3^ab^ ± 38.0	45.1^a^ ± 32.4	49.7^ab^ ± 34.2	<0.001	0.199	<0.001	<0.05
Successful (n)	28.9 ± 24.0	67.7 ± 42.0	51.8 ± 39.9	44.8 ± 38.8	77.0 ± 35.1	19.6 ± 13.7	61.6^B^ ± 53.3	44.9^A^ ± 36.6	42.1^A^ ± 31.4	44.6^A^ ± 31.7	<0.001	0.232	<0.001	<0.001
Failed (n)	2.34 ± 2.57	3.66 ± 3.69	3.53 ± 3.53	2.47 ± 2.84	3.75 ± 3.76	2.25 ± 2.41	0.56^A^ ± 0.73	3.40^BC^ ± 3.50	3.00^B^ ± 2.10	5.10^C^ ± 3.90	0.142	0.184	<0.10	<0.001
Successful (%)	89.5 ± 12.3	93.9 ± 5.87	92.2 ± 7.73	91.3 ± 11.6	94.8 ± 4.86	88.6 ± 12.3	98.2^B^ ± 4.91	91.8^AB^ ± 7.50	91.8^AB^ ± 5.30	85.0^A^ ± 14.2	<0.01	0.344	<0.001	<0.001
Failed (%)	10.5 ± 12.3	6.11 ± 5.87	7.84 ± 7.73	8.74 ± 11.6	5.16 ± 4.86	11.4 ± 12.3	1.80^A^ ± 4.91	8.20^AB^ ± 7.50	8.20^AB^ ± 5.30	15.0^B^ ± 14.2	<0.01	0.344	<0.001	<0.001
Landings per day	125 ± 97.8	286 ± 163	221 ± 162	189 ± 155	323 ± 126	87.5 ± 58.0	-	-	-	-	<0.001	0.178	<0.001	-
Successful (n)	116 ± 94.2	271 ± 159	207 ± 158	179 ± 150	308 ± 122	78.5 ± 54.7	-	-	-	-	<0.001	0.150	<0.001	-
Failed (n)	9.38 ± 4.98	14.6 ± 9.26	14.1 ± 7.61	9.88 ± 7.62	15.0 ± 9.41	9.00 ± 4.14	-	-	-	-	0.167	0.403	0.376	-
Successful (%)	87.4 ± 8.50	93.9 ± 3.37	91.4 ± 4.62	90.0 ± 9.21	95.2 ± 2.58	86.2 ± 7.37	-	-	-	-	<0.01	0.245	<0.001	-
Failed (%)	12.6 ± 8.50	6.11 ± 3.37	8.63 ± 4.62	10.0 ± 9.21	4.83 ± 2.58	13.8 ± 7.37	-	-	-	-	<0.01	0.245	<0.001	-

1 Early, observations from 5:00 to 8:00; Midday: observations from 9:00 to 12:00; Afternoon: observations from 13:00 to 16:00; Late: observations from 17:00 to 21:00. Data were collected from video recordings during 10 min, every hour from 5:00 until 21:00, 1 d/wk.

a,b Means with different superscript letters are different (*P* < 0.05).

A,B Means with different superscript letters are different (*P* < 0.01).

**Table 5:** Effect of age, enrichment with additional perches, genotype, and observation time on take-offs from the floor per observation time hens at housing in a cage-free aviary system (means ± SD).

	Week of age (A)		Additional perches (P)		Genotype (G)		Observation time^1^ (T)				*P* value			
Variables	17	20	No	Yes	W	B	Early	Midday	Afternoon	Late	A	P	G	T
Observations (n)	32	32	32	32	32	32	16	16	16	16				
Take-offs per observation time	20.1 ± 19.3	64.2 ± 61.7	40.3 ± 51.3	44.0 ± 50.5	74.3 ± 54.2	10.0 ± 11.0	69.1^B^ ± 79.5	32.9^A^ ± 39.5	30.2^A^ ± 30.3	36.4^A^ ± 29.5	<0.001	0.535	<0.001	<0.001
Successful (n)	19.8 ± 19.2	64.0 ± 61.7	39.9 ± 51.2	43.8 ± 50.5	73.9 ± 54.2	9.81 ± 10.91	68.8^B^ ± 79.5	32.7^A^ ± 39.4	30.1^A^ ± 30.2	35.9^A^ ± 29.5	<0.001	0.519	<0.001	<0.001
Failed (n)	0.31 ± 0.59	0.19 ± 0.40	0.34 ± 0.55	0.16 ± 0.45	0.31 ± 0.59	0.19 ± 0.40	0.25 ± 0.45	0.19 ± 0.4	0.13 ± 0.34	0.44 ± 0.73	0.291	0.120	0.291	0.474
Successful (%)	93.6 ± 20.0	99.7 ± 0.78	95.5 ± 18.0	98.4 ± 6.32	99.4 ± 1.38	94.0 ± 20.0	97.5 ± 8.29	92.5 ± 26.6	99.7 ± 0.81	97.7 ± 4.18	0.464	0.040	0.502	0.956
Failed (%)	6.39 ± 20.0	0.32 ± 0.78	4.49 ± 18.0	1.55 ± 6.32	0.63 ± 1.38	6.02 ± 20.0	2.46 ± 8.29	7.51 ± 26.6	0.31 ± 0.81	2.26 ± 4.18	0.464	0.040	0.502	0.956
Take-offs per day	80.4 ± 74.2	257 ± 204	161 ± 179	176 ± 182	297 ± 162	40.0 ± 33.0	-	-	-	-	<0.001	0.399	<0.001	-
Successful (n)	79.1 ± 73.9	256 ± 204	160 ± 178	175 ± 182	296 ± 162	39.3 ± 33.2	-	-	-	-	<0.001	0.3686	<0.001	-
Failed (n)	1.25 ± 0.71	0.75 ± 0.89	1.38 ± 0.74	0.63 ± 0.74	1.25 ± 0.89	0.75 ± 0.71	-	-	-	-	0.223	0.081	0.223	-
Successful (%)	93.3 ± 11.3	99.5 ± 0.68	97.3 ± 3.64	95.4 ± 11.6	99.4 ± 0.48	93.4 ± 11.4	-	-	-	-	0.156	0.645	0.169	-
Failed (%)	6.75 ± 11.31	0.51 ± 0.68	2.67 ± 3.64	4.58 ± 11.6	0.61 ± 0.48	6.64 ± 11.39	-	-	-	-	0.156	0.645	0.169	-

1 Early, observations from 5:00 to 8:00; Midday: observations from 9:00 to 12:00; Afternoon: observations from 13:00 to 16:00; Late: observations from 17:00 to 21:00. Data were collected from video recordings during 10 min, every hour from 5:00 until 21:00, 1 d/wk.

A,B Means with different superscript letters are different (*P* < 0.01).

The potential improvements in navigation skills during the early stages of the adaptation period are poorly investigated. In fact, most of the studies compared animal behavior, falls, and collision occurrence between arrival (19–20 wk) and 43 to 45 wk of age (Stratmann et al., 2015a,b, 2019), and they associated the reduction of collisions with the decreased activity of hens as age increased (Stratmann et al., 2019). On the other hand, over the first 4 wk after housing, our results revealed that navigation activity significantly improved: the number of landings and take-offs, and the rate of successful displacements increased, suggesting that young hens soon adapted to the new aviary system.

To favor navigation in the aviary, the introduction of additional structures, such as ramps, platforms, and perches, has been investigated during the last years both during the rearing of pullets, to train them to vertical movements, both in the laying phase, to facilitate vertical displacements within the different levels of the cage-free systems (EFSA AHAW Panel (EFSA Panel on Animal Health and Animal Welfare), 2015). Under our conditions, additional perches did not improve navigation ability of hens at housing. In fact, no significant difference in the number or in the rate of success of landings from any part/equipment of the aviary or the wall to the floor was recorded between enriched and not enriched pens (Table 4). This result was confirmed by the logistic regression analysis: the lack of additional perches did not result as a potential risk factor affecting failed landings (OR: 1.29; *P* > 0.05) (Table S6).

According to the literature, access to perches during rearing of pullets could positively affect their cognitive and physical abilities to use perches in the laying environment (Wichman et al., 2007). However, perches are considered also a cause of injury (Sandilands et al., 2009), and they can have a significant role in the development of keel deviations (Pickel et al., 2011; Harlander-Matauschek et al., 2015; O'Connor et al., 2015). In fact, housing systems containing perches can have a greater prevalence of keel bone fractures; perch height has also been positively correlated with the prevalence of fractures, suggesting the influential role of perches in collisions (EFSA AHAW Panel (EFSA Panel on Animal Health and Animal Welfare), 2015; Stratmann et al., 2015b). On the other hand, keel deviations and fractures have also been related to the shape of egg deposition rate, the egg production level, and the age at the first egg (Toscano et al., 2020).

Under our conditions, the univariate analysis also identified the lack of perches on the outer walls as a potential risk factor (Table S2): higher odds of failed landings were found when hens flew toward outer walls with only wooden boards, compared to walls with additional perches (OR: 2.65; *P* < 0.001). This result confirms that perches better suit to the flying ability of hens compared to wooden boards (EFSA AHAW Panel (EFSA Panel on Animal Health and Animal Welfare), 2015). Then, the odds of failed landings were greatly higher when comparing long with medium flight distance (i.e., hens starting from the third vs. the second tier of the aviary) (OR: 31.1; *P* < 0.001); odds were lower comparing short (i.e., hens starting from the first tiers of the aviary) with medium flight distance (OR: 0.17; *P* < 0.001) (Table 3).

The navigation activity and ability of pullets were largely different between the 2 genotypes, as Brown hens had higher odds of failed landings compared with White hens (OR: 6.65; 95% CI: 4.36–10.1) (Table 3). In fact, White hens performed a significantly higher number of landings in the observation interval (80.7 vs. 21.9; *P* < 0.001) with a higher success rate (94.8 vs. 88.6%; *P* < 0.001) compared to Brown hens (Table 4), as well as a higher number of take-offs (74.3 vs. 10.0; *P* < 0.001) without significant differences in the success rate (99.4 vs. 94.0%; *P* > 0.05) (Table 5).

Differences in navigation ability between genotypes have been reported also in previous studies in pullets (Chew et al., 2021) and laying hens (Scholz et al., 2014). At 4 wk of age, White (Lohmann Selected Leghorn) hens showed a higher number of successful jumps from the floor to the drinker and a lower number of failed landings from the drinker to the floor compared with Brown counterparts (Chew et al., 2021). A similar pattern was also observed for jumps from the floor to the ramp, whereas failed landings were not affected by the genotype (Chew et al., 2021). Brown strains (Lohmann Brown, Lohmann Tradition) of laying hens showed a higher rate of failed landings compared with white strains (Lohmann Selected Leghorn) (Scholz et al., 2014). This result could be related to a higher body weight and, therefore, to a higher wing load of Brown strains, which might have more difficulties to perform downward jumps, compared to the lighter White lines (Scholz et al., 2014). Such difference in live weight was also recorded in our trial (Table S1).

Under our conditions, navigation activity and ability were also evaluated at different time intervals during the day, that is, early (5–8 h), midday (9–12 h), afternoon (13–16 h), and late (17–21 h). Both for landings (62.2, 48.3, 45.1, and 49.7; *P* < 0.05) (Table 4) and take-offs (69.1, 32.9, 30.2, and 36.4; *P* < 0.001) (Table 5), the highest average number of displacements per observation time was recorded in the early observations, likely because of the activity following the switching on of the light in the barn. On the other hand, the rate of successful landings was the highest at the early hours (98.2%), the lowest at the late hours (85.0%), with intermediate results in the midday and afternoon observation times (91.8%) (*P* < 0.001) (Table 4). These results were confirmed by the odds ratio calculated by the multivariate logistic regression analysis (Table 3). Likely, the best results of the early hours can be associated with the absence/low occurrence of animals on the floor at the switch on the light when animals move for feed and water after the dark hours. On the other hand, the worst results of the late observations could be associated with the contemporary and quick recovery of all animals on the uppermost levels/perches of the aviary, the competition among them for these areas, and the falls of the animals from the aviary caused by overcrowding and competition.

The effects of the time of the day on falls still remain poorly understood, likely because of the possible interactions among several ontogenetic and management factors (e.g., genotypes, age of hens, light management, aviary design). In fact, contrasting results were reported in previous findings. In some cases, the likelihood of a collision was higher after lights on compared with the dusk phase and after lights off, which was associated with a higher activity of the animals during the day (Stratmann et al., 2019). In other studies, falls were mainly observed during dusk and after lights off (Stratmann et al., 2015a). On the other hand, Moinard et al. (2004) did not observe any effect of light intensity and light type on failed landings associated with downward or upward jumps.

In our study, the significant interactions observed between genotype and week of age for the number of landings (53.5 and 8.94 in White and Brown hens at 17 wk of age; 108 and 34.8 in White and Brown hens at 20 wk of age; *P* < 0.001) (Table S4a) and the number of take-offs per observation time (37.1 and 3.13 in White and Brown hens at 17 wk of age; 111 and 16.9 in White and Brown hens at 20 wk of age; *P* < 0.001) (Table S5a) further stress the large differences in navigation activity between the 2 genotypes at housing and after 4 wk. Brown hens undoubtedly moved less and likely used a different pattern of movements among the different levels of the aviary, possibly jumping level by level, compared to White hens. On the other hand, in our trial, differences between the 2 genotypes in navigation activity and ability were not associated with differences in the occurrence of keel lesions at 20 wk of age (only 2 Brown hens from pens without additional perches showed keel deviations, 0.25% of controlled animals) (Table S1). Nevertheless, this result is not conclusive as occurrence of keel lesions has to be measured at the end of the laying period due to the relationships between egg production level, deposition curve, and keel bone lesions (Toscano et al., 2020), and possible interactions with equipment of the aviary.

## CONCLUSIONS

Facilitating adaptation and navigation ability of pullets at housing in the new facilities for the laying phase is expected to play a positive effect, both on animal welfare and production results, especially when cage-free systems are used. Under the condition of the present study, the enrichment with additional perches on an enriched outer wall did not improve navigation activity and ability of laying hens at housing, but long-term effects on space use and competition for resources should be evaluated over the laying period. The 2 tested genotypes exhibited substantial differences as regards the use of space and additional perches, as well as the navigation activity and ability, since the first week after housing, with White hens showing a preference for additional perches, a less homogeneous distribution and space use, and more displacements. The uneven use of space and resources could increase competition for specific areas/equipment and, thus, produce abnormal behaviors and stress to animals. Differences in navigation skills, related to the hen ability of safely and freely access the different resources, can also largely affect the behavior and budget time of laying hens. Thus, welfare and production results should be included for a complete evaluation with reference to the factors tested in the present trial (additional perches, genotypes) and, in perspectives, different equipment (e.g., ramps), designs, and arrangements (e.g., position, available linear space per hen), to optimize cage-free systems.

## Appendix Supplementary materials

Image, application 1

## Footnotes

Supplementary material associated with this article can be found in the online version at doi:10.1016/j.psj.2023.102962.
